# Does parental angling selection affect the behavior or metabolism of brown trout parr?

**DOI:** 10.1002/ece3.7220

**Published:** 2021-02-03

**Authors:** Jenni M. Prokkola, Nico Alioravainen, Lauri Mehtätalo, Pekka Hyvärinen, Alexandre Lemopoulos, Sara Metso, Anssi Vainikka

**Affiliations:** ^1^ Department of Environmental and Biological Sciences University of Eastern Finland Joensuu Finland; ^2^ Natural Resources Institute Finland (Luke) Kainuu Fisheries Research Station Paltamo Finland; ^3^ Department of Biology University of Turku Turku Finland; ^4^ Present address: Organismal and Evolutionary Biology Research Programme University of Helsinki Helsinki Finland

**Keywords:** animal personality, fishing, respirometry, *Salmo trutta*

## Abstract

The behavior of organisms can be subject to human‐induced selection such as that arising from fishing. Angling is expected to induce mortality on fish with bold and explorative behavior, which are behaviors commonly linked to a high standard metabolic rate. We studied the transgenerational response of brown trout (*Salmo trutta*) to angling‐induced selection by examining the behavior and metabolism of 1‐year‐old parr between parents that were or were not captured by experimental fly fishing. We performed the angling selection experiment on both a wild and a captive population, and compared the offspring for standard metabolic rate and behavior under predation risk in common garden conditions. Angling had population‐specific effects on risk taking and exploration tendency, but no effects on standard metabolic rate. Our study adds to the evidence that angling can induce transgenerational responses on fish personality. However, understanding the mechanisms of divergent responses between the populations requires further study on the selectivity of angling in various conditions.

## Graphical abstract

Angling‐induced divergent responses on a wild and a hatchery population of brown trout, making hatchery population juveniles from low‐vulnerability parents more explorative than offspring from high‐vulnerability parents, but having no effect on juveniles from the wild population. Our results imply that angling selection may lead to accumulating behavioral changes across generations. Whether this will manifest as increased shyness, as has been predicted, can depend on the background of the population or the ecological conditions during angling.

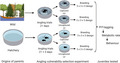

## 1 INTRODUCTION

Survival selection by hunting and fishing can differ from natural selection patterns and induce phenotypic changes in various traits over time (Fugère & Hendry, 2018). At worst, anthropogenic selection can increase the relative frequencies of maladaptive phenotypes decreasing the fitness of harvested populations (Allendorf & Hard, 2009; Coltman et al., 2003). Experimental studies have shown that responses to human‐induced selection can be rapid at both phenotypic, including behavior (Kern et al., 2016; Sbragaglia et al., 2019; Wong et al., 2012) and genetic levels (Bowles et al., 2020; Cooke et al., 2007; Sutter et al., 2012; Uusi‐Heikkilä et al., 2015). Fisheries‐induced selection can occur on traits that explain vulnerability to fishing and on traits that enable the fish to reproduce before becoming captured (Cooke et al., 2007; Hollins et al., 2018; Redpath et al., 2010; Sutter et al., 2012; Uusi‐Heikkilä et al., 2008).

In the context of recreational fisheries, selection is predicted to act mainly on behavior, as the vulnerability to being captured depends on fish behavior, and capture leads to either survival costs or other nonlethal fitness costs (Lennox et al., 2017; Uusi‐Heikkilä et al., 2008). Vulnerability to capture by passive fishing gear, including angling, depends on traits related to risk taking and curiosity, such as boldness and exploration tendency (Arlinghaus et al., 2017; Cooke et al., 2007; Härkönen et al., 2014; Wilson et al., 2015, reviewed in Lennox et al. (2017)), although not all studies have supported the predicted role for boldness (Louison et al., 2017; Vainikka et al., 2016). Over time, angling selection could increase the frequency of shy phenotypes in the population (Alioravainen et al. (2020); Andersen et al., 2018; Arlinghaus et al., 2017). The shift in the behavior of fish populations may occur on top of the fishing/angling‐induced decrease of body size and age‐at‐maturity (Bowles et al., 2020; Sharpe & Hendry, 2009) and cause a personality‐related decrease in resource acquisition. Eventually, these can lead to complex effects on stock recruitment (Arlinghaus et al., 2017). However, these predictions for the existence and consequences of increasing shyness due to passive fishing gear require further empirical tests.

Selection acting on personality could induce correlated metabolic effects due to physiological covariation between behaviors affecting energy balance and standard metabolic rate (SMR) (e.g., Killen et al., 2011; Mathot et al., 2018). In one of the first empirical angling selection studies, SMR was found to be 10% lower in a low‐vulnerability selection line compared to a high‐vulnerability selection line in largemouth bass (*Micropterus salmoides*) (Redpath et al., 2010). However, several studies have found no phenotypic association between angling vulnerability and SMR (Väätäinen et al., 2018), or between angling vulnerability and several metabolic traits, including SMR (Louison et al., 2017, 2018). Given that metabolic traits are also sensitive to environmental conditions, and angling methods may impose different selection pressures in different experiments, the potential for evolutionary response in metabolic traits in response to angling‐induced selection is presently not well understood.

Salmonids, just as many other taxa, can display individually distinctive behavioral strategies and coping styles (Adriaenssens & Johnsson, 2011; Brelin et al., 2008; Huntingford & Adams, 2005; Näslund & Johnsson, 2016; Vindas et al., 2017), on which selection may act. Due to their widespread hatchery rearing, species such as the brown trout are also affected by unintended domestication, introducing shifts in life‐history traits, behavior (Araki et al., 2008; Horreo et al., 2018; Huntingford, 2004), and vulnerability to angling (Klefoth et al., 2013); more research on the effect of unintended domestication on fish populations used in supplemental stocking is therefore warranted.

Here, our goal was to test whether angling could induce selection in behavioral traits measured under authentic predator cues or in SMR by studying one‐year‐old offspring of brown trout (*Salmo trutta*) from both captive and wild origins. By using replicated behavioral assays involving predator cues and collecting metabolic rate data, we add to the study by Alioravainen et al. (2020), which focused on open‐field tested personality of the offspring from the same angling experiments during their first summer. We further compared fish acclimated under 12 hr light:12 hr dark or 24 hr light, because unnatural light conditions may be perceived as stressful by the fish and modify the phenotypic responses. We hypothesized that offspring from angling‐vulnerable parents would have higher scores in risk‐taking behavior, and higher SMR, compared to fish from nonvulnerable parents in both strains of fish.

## 2 MATERIAL AND METHODS

### 2.1 Angling experiment and fish husbandry

Experiments were carried out between 2015 and 2017 at the Kainuu Fisheries Research Station (www.kfrs.fi) of Natural Resources Institute Finland (Luke) under ethical license obtained from the national Animal Experiment Board in Finland (license number ESAVI/3443/04.10.07/2015). We used (a) wild, predominantly nonmigratory, parental brown trout from River Vaarainjoki captured by electrofishing, and (b) captive (5–6th generation), predominantly migratory brown trout from so‐called Lake Oulujärvi hatchery strain. The founders of the captive brood stock came from two source populations, River Kongasjoki and River Varisjoki (Alioravainen et al., 2020; Lemopoulos, Prokkola et al., 2019). Despite originating from the same River Varisjoki watershed, only a few km apart, the captive and *R*. Vaarainjoki populations showed moderate genetic divergence based on fixation index (*F*_ST_ *‐*value) of 0.11 (Lemopoulos, Prokkola et al., 2019). Both populations had experienced fishing pressure (mainly hook‐and‐line fishing) in the past, but not since R. Vaarainjoki was protected from fishing in the 1990s and the original captive population was established in the 1960s–1980s.

**FIGURE 1:**
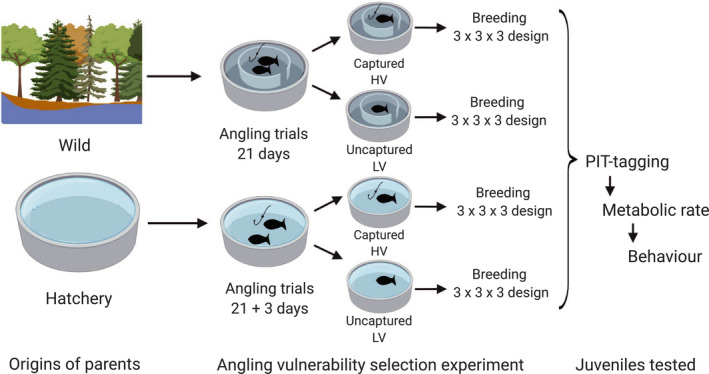
A diagram of the experimental design. Parental fish were placed in size‐assortative ponds (*N* = 64 and 167 for large‐ and small‐size fish in hatchery population, and *N* = 53 and 91 in the large‐ and small‐size wild fish, respectively) before angling. After 21 days of angling, or after the first capture, fish were transferred to two tanks similar to those used in the angling trials. The hatchery stock individuals were thereafter fished for a second time (3 days of angling). Before breeding, fish were again combined in population‐specific size‐assortative ponds (not shown in diagram). Further details in Alioravainen, Hyvärinen et al. (2020)

In 2015, hatchery‐origin and wild‐origin adult fish were exposed to experimental fly fishing and divided into captured (high vulnerability, HV) and uncaptured (low vulnerability, LV) groups (Figure 1) (Alioravainen, Hyvärinen et al., 2020). Fish were fished in two size‐assortative pools for each population during June and July with fly fishing gear adjusted by the size of the fish in the pools. The wild fish were fished in seminatural 50‐m^2^ ponds with a gravel‐bottom outer riffle sections and *ca*. 1 m deep, concrete inner pool sections (53 and 91 visually size‐sorted fish in two ponds). The hatchery fish were fished in 75‐m^2^ concrete ponds with no structures (64 larger and 167 smaller fish from two different cohorts in two ponds). Angling was performed by experienced fly fishers (mainly A.V.) using unnaturally colored woolly bugger‐type fly patterns tied to barbless hooks. During angling sessions, an angler fished a pond until a fish took the fly or five minutes passed, after which angling was continued at earliest one hour later. If a fish was captured, angling was continued immediately after processing, which included anesthesia with benzocaine (40 mg/L), identification of passive integrated transponder (PIT) tag (Oregon RFID), or tagging when a pre‐existing tag was missing, and measuring total length (to 1 mm) and weight (to 2 g). Fish that were missing PIT tags were tagged under the skin next to the dorsal fin using 12 mm tags at this point. After processing, the fish were transferred to similar ponds (hatchery fish to a 50‐m^2^ otherwise similar concrete pond) as used for each population during angling. After angling trials were finished, on June 25, 2015, all remaining wild fish that were not captured were collected by dip‐netting after draining the experimental angling ponds, anesthetized, measured, and weighed (mean body lengths of fish uncaptured and captured by angling: in large fish 457 and 475 mm, respectively, and in small fish 344 and 354, respectively). Uncaptured wild fish were then combined in the same ponds as the fish captured by angling. The captured hatchery strain fish were subjected to a second round of angling ~2 weeks later to identify the most vulnerable fish, where in total eight fish were captured and prioritized for breeding the highly vulnerable line, but this was not done on wild fish due to their limited availability. Angling trials finished on July 8, 2015, and also hatchery fish were transferred back to their original ponds. Because of the warm water at the time of finishing the second round of angling, the uncaptured hatchery fish were not measured to avoid handling‐induced stress and mortality. One deep‐hooked small hatchery fish was found dead 5 days and one large hatchery fish 41 days after capture, but otherwise, no mortality occurred between angling trials and the breeding.

From the hatchery stock, 32.8 and 50.9% (corresponding to 21 and 85 fish), and from the wild stock, 28.3 and 24.2% (corresponding to 15 and 22 fish) of large and small individuals were captured, respectively. Notably, the wild fish had natural invertebrate food available in their ponds, and the structured ponds offered more hiding places, and they had clearly lower catchability than the hatchery fish (Alioravainen, Hyvärinen et al., 2020). Very few wild fish were captured in one angling session (maximum 4) compared to the hatchery fish (maximum 11). The captured and noncaptured parent fish were similar in size, indicating that vulnerability to angling was most likely related to size‐independent traits (Alioravainen, Hyvärinen et al., 2020).

The offspring used in this study were obtained from fish bred in four groups (i.e., high and low vulnerability [HV and LV, respectively] within each population) in the autumn of 2015. Parent fish from large‐ and small‐size groups were mixed for breeding. A replicated, fully factorial 3 × 3 breeding design was used to create the F_1_‐generation; males were crossed with females in all combinations in one matrix, and the matrices replicated three times for each group, details in Appendix S1, and in Alioravainen, Hyvärinen et al. (2020). In the autumn of 2016, the one‐summer‐old fish were tagged with individual 12‐mm PIT tags in the abdominal cavity under anesthesia (benzocaine). After tagging, the selection lines were mixed together in two 3.2 m^2^ fiberglass rearing tanks. During the whole study, fish were fed with commercial fish pellets (Raisio Oyj).

### 2.2 Photoperiod acclimations

In mid‐March 2017, after being reared under constant light, 100 fish were divided into two different photoperiod treatments in 0.4‐m^2^ green, plastic, flow‐through tanks. The tanks were covered with green nets. One treatment continued to be reared under constant light (approximately 9 lux at the water surface, *n* = 10/selection line/population, 40 fish in the tank), and the second treatment received a 12 hr light:12 hr dark (L:D) acclimation (approximately 12 lux during light period at the water surface, *N* = 15/selection line/population divided equally in two tanks, details in Appendix S1).

Fish were fed using automatic belt feeders (~0.3% fish mass per day) on 5–6 days per week during approx. 4 hr between 8:00 and 20:00 to avoid the entrainment of endogenous rhythms by feeding. After a minimum two‐week acclimation, the metabolic rate measurements were started.

### 2.3 Measurement of standard metabolic rate

The SMR, that is, postabsorptive, resting O_2_ uptake ($\dot{M}$O_2_) (Nelson, 2016), of fish was measured using intermittent flow‐through respirometry (Svendsen et al., 2016). The fish were not fed for 40–48 hr before the start of the measurement to minimize the effect of digestion on metabolic rate. As fish from the same tank were measured on multiple days, fish in each tank were fed on a rotation of 40–48 hr fasting followed by 1.5 days of feeding. The fish were caught by dip‐netting under a dim red light into 10‐L buckets, identified with a PIT‐reader and transferred to the flow‐through measurement chambers (diameter 33 mm, length 120 mm, Loligo Systems, Viborg, Denmark). The chambers were immersed in a water bath, which was also immersed in a flow‐through tank. Measurements were started immediately and continued for approximately 23 hr, corresponding to 90–96 15–17‐min measurement cycles for all individuals. We measured 2–4 individuals in separate horizontal glass chambers during each day of measurements. Oxygen saturation was measured in % of air saturation using two‐point‐calibrated DAQ‐PAC‐WF4 system with Sensor spot mini sensors and recorded every second in AutoResp software (Loligo Systems). Water temperature was measured using the Pt1000 temperature probe placed in the respirometer tank (Loligo Systems). Air pressure in kPa, with one decimal, was recorded daily at the start of measurements from a nearby weather station.

The respirometry measurements were started between 11:45 a.m. and 12:10 p.m. by measuring the oxygen level in each empty chamber for one cycle to establish a baseline for bacterial oxygen consumption. The cycles consisted of a 6‐min flush and a 9–11‐min recirculation period, including a 5.5–7.5‐min wait period to allow mixing of the water and a 3.5‐min measurement. The temperature in the acclimation tanks was on average 3.4°C ± *SD* 0.12°C during the respirometer measurements, but the respirometer temperature was slightly higher than the acclimation temperature (range 3.4–4.2°C) due to the unavoidable heating of the tank by the measurements. During the measurement, the respirometer was covered with a green net, similar to what was used to cover the acclimation tanks prior to measurements, and disturbances were kept to a minimum. The fish were passive for extended periods of time during the measurements (observed from oxygen measurements). The chambers were washed using mild Deconex disinfectant, and the water inside the respirometer tank was changed every 5–7 days during the measurements. Six measurements, where air bubbles were observed in the measurement chamber, were discarded from the analysis. The photoperiod was 12 hr:12 hr L:D during respirometry for all fish, because the lowest consumption was expected to occur during the dark phase.

After measurements, fish were anesthetized with benzocaine, measured for total length (to 1 mm), and weighed (to 0.1 g), after which they were transferred to new 0.4 m^2^ tanks similar to those used prior to measurements. The fish were under the same photoperiod as before the measurements and fed daily at varying times. After removing the fish, respirometer chamber oxygen levels were measured empty for one cycle to quantify bacterial respiration rates. No measurable respiration was detected without fish. The slope of the decrease in oxygen level during each 3.5‐min measurement period was calculated using linear regression with FishResp (Morozov et al., 2019) in R. An initial acclimation period was excluded by including only measurements taken after 16:00 in the analysis. We accepted all slopes where the *R* ^2^ was > 0.9 in the calculation: This resulted in 12–70 slopes being accepted for each individual, after data from one individual with only 7 accepted slopes were excluded. R code for analyzing the raw data is provided in GitHub (see *Data availability*).

The SMR was calculated as the average $\dot{M}$O_2_ across all accepted cycles after comparing data obtained using several methods following Chabot et al. (2016): quantile 0.1, quantile 0.2, lowest 10 consumption values, and average. The average of accepted cycles was used as it was the only variable that showed a positive mass dependence of metabolic rate. Other variables were not correlated with fish body mass, likely indicating inconsistencies in fish‐specific accepted measurements due to variation in *R* ^2^ values. The average of accepted cycles represents fish in postabsorptive resting conditions, excluding initial handling stress period, and thus, we expect it to be close to the real SMR of the fish.

### 2.4 Setup of behavioral trials

**FIGURE 2:**
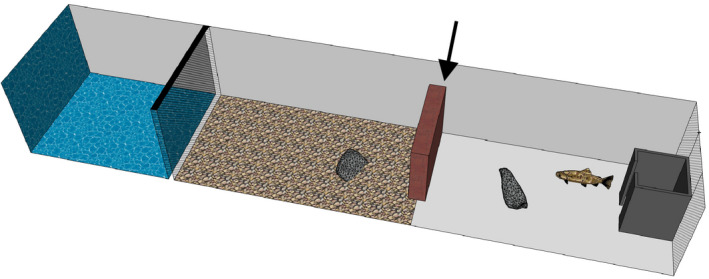
A 3D‐illustration of the arena used in personality trials without the left side wall. Water flow direction is left–right. Burbot was placed in the area indicated by blue color, upstream from the net (inaccessible to the brown trout). Pebbles were scattered in the brown‐colored area. The grey box indicates the start box, where the fish was placed before the start of a trial. Latency was measured as time to emerge from the box. Exploration intensity was measured as swimming activity outside the start box arena after emergence. Exploration tendency was measured as whether or not the whole body of fish passed the gate indicated by an arrow

The fish were allowed to recover from respirometry for at least four days before assayed for behavioral traits under chemical predator cues. They were not fed for 24‐hr prior to behavioral trials. The trials were conducted in custom‐made mazes (Figure 2) (size 400 mm wide × 1,500 mm long, water depth 100 mm in the open area). During the trials, temperature in the maintenance tanks and test arenas was on average 5.0 ± *SD* 1.5°C. The rate of water flow was adjusted to ~8 L/min (~7.6–8.8 L/min) during the trials. This allowed for at minimum 1.26 times the arena volume of water to flow between consecutive trials, which was considered sufficient to minimize potential carry‐over effects of chemical cues between trials. The arena was lit by LED lights (CRI90 LED chain in waterproof silicon tube, 3000–3300K, 4.8W m^2^) situated along one long edge of the arena (>70 lux across the arena depending on the distance from the light source). Half‐way across the arena was a brick gate situated next to one side, allowing entry from the other side. Behind the brick, natural pebbles (~3–5 cm in diameter) were scattered unevenly on the floor. One stone was provided for shelter, and another was in the center of the arena in front of the start box (Figure 2). Four structurally similar arenas were used in the experiment, but two of the arenas were mirror images of the other two with respect to the location of the gate.

Upstream from the flow‐through test arena section was a section divided by a metal grid (5 mm mesh size) where a hatchery‐reared burbot (*Lota lota*) (length ~30–40 cm) was placed to introduce chemical cues of a natural predator of juvenile brown trout. Burbot are nocturnal bottom‐dwelling predators that are likely difficult for prey to detect visually, but their odor induces antipredator responses in prey species (Ylönen et al., 2007). Burbot were maintained by feeding them with pieces of various cyprinids and vendace (*Coregonus albula*) during rearing, but fresh pieces of brown trout were used for two days prior to and every 2–3 days during the trials (feeding occurred in separate tanks, not the behavioral arenas). Approx. 5g of brown trout /meal was offered to the burbot, but not all pieces were eaten presumably due to low temperature. Burbot (*N* = 8) were moved to the test arenas at least one day before the trials and changed in each arena every 10–15 trials (2–3 days).

Before each trial, individual brown trout were haphazardly dip‐netted from their rearing tanks under red light and placed into black 10‐L buckets filled with ~8 L of water from the flow‐through system. Fish were identified by PIT tags and left undisturbed for 10 min before being transferred into the start box located downstream from the test arena by pouring. During each trial, the trout was acclimatized in the start box for 3 min, after which the door of the box was opened by pulling a string from behind a curtain. The movements of the fish were recorded from above using two CCTV infrared cameras (two arenas simultaneously filmed using the same camera) for 10 min (of which the first 9 min 45 s was included in the behavior analysis). The behavioral trial was repeated three times between 8:00 and 11:00 for each focal fish, with an average time of 4.3 days (range 1–8 days) between consecutive trials. One trial from four fish was omitted from the analysis due to error in data collection. The order in which batches of four fish were captured on the same day from the same tank for the four arenas was recorded (batch from hereon, levels 1–5, four individuals from batch 6/7 combined to batch 5).

### 2.5 Testing behavioral responses to burbot

To confirm that burbot odor was perceived as risky by the brown trout in the personality assays, we studied the response of brown trout to burbot odor in a separate experiment using individuals from wild HV (*N* = 9) and wild LV groups (*N* = 10). These fish were acclimated in similar tanks as the personality‐tested fish at 12 hr:12 hr L:D photoperiod for one week. The behavior of each individual was tested on six different days in the presence and absence of predator odor (3 trials in each condition in haphazard order). 3–4 different arenas were used for each fish on different days to reduce fish habituation to a certain arena. All trials were conducted between 14:40 and 17:00. Control treatment arenas were emptied and thoroughly rinsed with pressurized tap water and water flow maintained for >2 hr before the trials to avoid carry‐over effects from burbot odor in previous trials (it was not possible to conduct the tests prior to the experiment on the angling selection lines due to the limited time period with stable water temperature in the hatchery). The water used in the flow‐through system originated from lake Kivesjärvi, where burbot is a common species; thus, dilute traces of burbot odor may have been present throughout the study.

### 2.6 Analysis of video recordings

Behavioral data were collected from videos using manual tracking with AV Bio‐Statistics 5.2 timing software. The observers were blind to the identity of fish in all recordings. Analyses were conducted in haphazard order, and each trial was analyzed once. In total, four people analyzed the videos (JMP, NA, AL, and SM). Four behaviors were characterized along the “exploration” personality axis, as defined by Réale et al. (2007). That is, fish were in a new environment during the tests and the odor of predator was previously unknown to the fish given the fish had not experienced natural predation risk. The behavioral events timed from the videos were (1) *latency*—the time from the start of the experiment until the whole body of fish emerged from the start box (after Boulton et al., 2014; Moran et al., 2016; Vainikka et al., 2016); (2) time until fish passed the gate to the upstream section of the arena (arrow in Figure 2), but this was not analyzed because of many fish not entering this section; instead, we recorded (3) *exploration tendency*—a binary variable indicating whether the whole body of the fish passed the gate within the arena; and (4) *exploration intensity*—the proportion of time spent actively swimming after emerging from the start box. We used the proportion of time rather than absolute time to reduce the dependence of activity from latency. Activity was thus calculated as the total mobile time outside the box divided by total time outside the box. Fish was considered immobile when not moving for longer than ~2 s.

### 2.7 Sex determination from DNA samples

To consider potential sex differences in the studied traits, we identified the sex of fish using PCR amplification of the sexually dimorphic *sdY* locus, which identifies the correct sex in brown trout with nearly 100% accuracy (Quéméré et al., 2014), details in Appendix S1.

### 2.8 Statistical analyses

A principal component analysis (PCA) of behavioral data collected from the fish that emerged from the box, that is, data with no missing observations showed that eigenvalues were all <1.5, that is, that the three variables were not strongly correlated (Appendix S1: Figure A1 and A2). Therefore, each behavioral variable was analyzed as separate response variable using univariate models (Table 1 for sample sizes).

**TABLE 1:** The number of individuals in each group in each analysis, mass‐specific SMR values, and fish total body length and mass (mean ± *SD*) at the end of the experiment. (HV = high and LV = low vulnerability)

Photoperiod	Population	Selection line	*N* (metabolic rate)	*N* (latency, exploration tendency & intensity)	*N* (body size)	*N* males/females (unknown)	Body length/mm	Body mass/g	SMR mg O_2_ L^−1^ kg^−1^ hr^−1^
12:12	Hatchery	HV	15	15	14	9/5 (1)	117 ± 10	17.1 ± 4.3	260.7 ± 65.5
		LV	10	14	14	6/2 (7)	115 ± 13	16.5 ± 6.7	300.4 ± 16.5
	Wild	HV	13	14	14	6/6 (3)	117 ± 7	17.4 ± 3.2	261.9 ± 41.7
		LV	15	15	14	7/7 (1)	115 ± 9	16.6 ± 4.3	268.8 ± 49.5
24	Hatchery	HV	9	10	9	6/3 (1)	119 ± 9	17.7 ± 4.7	261.2 ± 36.0
		LV	7	10	6	0/4 (6)	124 ± 9	19.6 ± 3.9	299.4 ± 59.4
	Wild	HV	8	10	6	2/3 (5)	116 ± 9	17.8 ± 7.1	260.9 ± 55.3
		LV	6	10	5	2/1 (7)	119 ± 9	17.8 ± 3.0	305.2 ± 71.8

Variation in each response variable (SMR and behavioral variables) was explained with a univariate model, including breeding group and acclimation conditions as explanatory terms (Table 2). All analyses were conducted in R v.3.3.2 (R Core Team, 2019). Linear (LMM) and generalized mixed‐effects models (GLMM) were fitted using package *lme4* (Bates et al., 2015) with *lmerTest* (Kuznetsova et al., 2017) and the frailty models using package *coxme* (Therneau, 2018). The data were visualized using ggplot2 (Wickham, 2009). Statistical significance was determined as *α* = 0.05 in all models.

**TABLE 2:** The main statistical models used in this study. Abbreviations explained below the table

Study section	Response variable	Model
I. Angling selection experiment	Log_10_ (SMR)	*y_ij_* *=* *β*_0_ *+* *β*_1_PHO*_ij_* *+* *β*_2_POP*_ij_* *+* *β*_3_SEL*_ij_* *+* *β*_4_POP*_ij_* *×* SEL*_ij_* *+* *β*_5_POP*_ij_* *×* PHO*_ij_* *+* *β*_7_logBM*_ij_* *+* *β*_8_WT*_ij_* *+* *p_l_* *+* *e_ij_*
	Exploration intensity	*y_ijk_* *=* *β*_0_ *+* *β*_1_PHO*_ijk_* *+* *β*_2_POP*_ijk_* *+* *β*_3_SEL*_ijk_* *+* *β*_4_POP*_ijk_* *×* SEL*_ijk_* *+* *β*_5_POP*_ijk_* *×* PHO*_ijk_* *+* *β*_6_REP*_ijk_* *+* *b_i_* *+* *c_j_* *+* *d_k_* *+* *e_ijk_*
	Latency	$\lambda \left(t\right)=\lambda_{0}\left(t\right)e^{\beta_{1}{\text{PHO}}_{\mathrm{ijk}}+\beta_{2}{\text{POP}}_{\mathrm{ijk}}+\beta_{3}{\text{SEL}}_{\mathrm{ijk}}+\beta_{4}{\text{POP}}_{\mathrm{ijk}}\times {\text{SEL}}_{\mathrm{ijk}}+\beta_{5}{\text{POP}}_{\mathrm{ijk}}\times {\text{PHO}}_{\mathrm{ijk}}+\beta_{6}{\text{REP}}_{\mathrm{ijk}}+b_{i}+c_{j}+d_{k}}$
	Exploration tendency (1 = explorative, 0 = un‐explorative)	*y_ij_* *~* Bernoulli(*p_ijk_*) logit(*p_ijk_*) *=* *β*_0_ *+* *β*_1_PHO*_ijk_* *+* *β*_2_POP*_ijk_* *+* *β*_3_SEL*_ijk_* *+* *β*_4_POP*_ijk_* *×* SEL*_ijk_* *+* *β*_5_POP*_ijk_* *×* PHO*_ijk_* *+* *β*_6_REP*_ijk_* *+* *b_i_* *+* *c_j_* *+* *d_k_*
II. Behavioral responses to burbot olfactory cues	Exploration intensity	*y_ijk_* *=* *β*_0_ *+* *β*_1_SEL*_ijk_* *+* *β*_2_TRE*_ijk_* *+* *β*_3_REP*_ijk_* *+* *β*_4_BL*_ijk_* *+* *b_i_* ^(1)^CON *+* *b_i_* ^(2)^BUR *+* *c_j_* *+* *d_k_* *+* *e_ijk_*
	Latency	$\lambda \left(t\right)=\lambda_{0}\left(t\right)e^{\beta_{1}{\text{SEL}}_{\mathrm{ijk}}+\beta_{2}{\text{TRE}}_{\mathrm{ijk}}+\beta_{3}{\text{REP}}_{\mathrm{ijk}}+\beta_{4}{\text{BL}}_{\mathrm{ijk}}+b_{i}+c_{j}+d_{k}}$
	Exploration tendency	*y_ijk_* *~* Bernoulli(*p_ijk_*) logit(*p_ijk_*) *=* *β*_0_ *+* *β*_1_SEL*_ijk_* *+* *β*_2_GR*_ijk_* *+* *β*_3_REP*_ijk_* *+* *b_i_* *+* *c_j_* *+* *d_k_*

Note

*β*_0_ Intercept, PHO Photoperiod, POP Population, SEL Selection, logBM Log_10_ body mass in kg, WT Water temperature in °C, BL Body length in mm – mean (118.8182 mm for I, 122.4464 mm for II), REP Trial repeat, TRE Treatment, *p*_l_ the random effect for chamber l, *b_i_* random effect for fish *i*, *c_j_* the random effect for arena *j*, *d_k_* the random effect for batch *k*, *e* Residual, *λ*_0_ baseline hazard, *t* time, CON/BUR binary explanatory variables for burbot and control treatments.

SMR and body mass were log_10_‐transformed for the LMM to account for the scaling of metabolic rate with body mass. The models used for different behavioral variables were as follows: (1) LMM for *exploration intensity*, where a higher value indicates higher proportion of time spent actively swimming, (2) a frailty model (i.e., mixed effect Cox proportional hazards models for time‐to‐event data (Collett, 2015)) for *latency*, where higher trait values and negative model coefficients indicate longer time to emergence, and (3) a GLMM for *exploration tendency* (Bernoulli‐distribution), where entering or not entering the upstream sector was indicated by 1 and 0, respectively. Trial repeats were encoded as a continuous variable: −1, 0, and 1 in data from angling selection experiment and as 1–6 from burbot versus control experiment. In 8 trials, the fish jumped out of the start box prior to the trial and their behavior was analyzed for 9 min 45 s minutes after the jump.

For LMMs and GLMM, the main effects of population, selection line, and photoperiod were separately tested using linear hypothesis testing (function *lht* in package *car*) using restricted models, where each respective main effect and its interactions were defined zero and compared to the full model using *F* tests. From LMM and GLMM models, the estimated marginal means and confidence intervals were estimated with package *ggeffects* (Lüdecke, 2018). All linear models were checked for homoscedasticity and normality of residuals. For all response variables, the effect of sex was analyzed in models including the fixed effect of sex as well as the effects from original models, except photoperiod or its interactions due to limited sample size with known sex. For further details, see Appendix S1 and *Data accessibility*.

To assess individual‐level correlation among the behavioral variables and SMR, Pearson's product‐moment correlations were calculated between residuals from a linear model with log_10_ SMR as the response variable and log_10_ body mass in kg as the explanatory variable, and Best Linear Unbiased Predictors (BLUPs) for latency or exploration intensity. BLUPS were obtained from linear mixed models including only individual as random effect, and only for the trials where the fish emerged from the start box.

## 3 RESULTS

### 3.1 SMR

**FIGURE 3:**
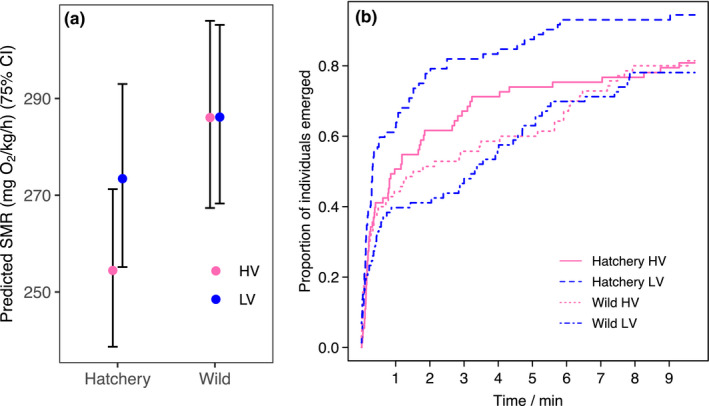
Predicted SMR (estimated marginal means) across two populations and angling selection lines (HV—high vulnerability and LV—low vulnerability) in brown trout. Data from two photoperiods combined. Estimates and 75% confidence intervals were obtained from a linear mixed model and conditioned on fish average body mass and average temperature during measurements, back‐transformed to linear scale and divided by average body size in kg to obtain mg O_2_ kg^−1^ hr^−1^. For N in each group, see Table 1

The LMM indicated a significant effect of body mass and population on SMR, with fish from wild background having higher SMR than fish from the hatchery background, but no effect of selection line (Figure 3, Table 3). Sex of fish had no effect on SMR (*F*_1, 58.215_ = 0.067, *p* = .797).

**TABLE 3:** Results from model for SMR. The zero levels for contrasts were as follows: photoperiod 12:12, population hatchery, and selection line HV

Fixed effects	Estimate ± SE	Num *df*	Res/Den *df*	F	*p*
Intercept	0.97 ± 0.20	1	74.522	4.96 (*t*)	<.001
Photoperiod	0.004 ± 0.02	2	72.09	0.41	.664
Population	**0.051 ± 0.02**	**3**	**73.355**	**2.78**	**.047**
Selection	0.031 ± 0.02	2	72.281	1.27	.286
Temperature	0.02 ± 0.02	1	73.866	1.32	.254
Log_10_ body mass	**0.27 ± 0.09**	**1**	**72.773**	**9.90**	**.0024**
Pop × selection	−0.031 ± 0.03	1	72.173	1.342	.250
Pop × photoperiod	0.014 ± 0.03	1	72.119	0.241	.625

Random effects	Variance (*SD* ^2^)
Chamber	0.039^2^
Residual	0.060^2^

Note

*F*‐ and *p*‐values for the interactions and temperature effect were obtained from Type III sums of squares and Satterthwaite approximation for degrees of freedom. For the other fixed effects, linear hypothesis tests using *F* test on restricted models with each main effect and its interactions set to zero were used—residual degrees of freedom are given for these tests. Significant (*p* < .05) effects shown in bold. For the intercept, *t* test value is shown.

### 3.2 Behavior in angling selection lines

**FIGURE 4:**
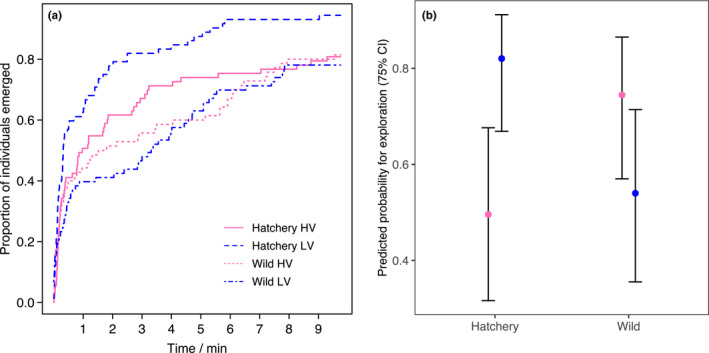
Behavioral differences between two angling vulnerability selection lines (HV—high vulnerability and LV—low vulnerability) within the hatchery and wild populations. Data from photoperiods combined for clarity. (a) Curves showing the proportion of individuals emerged from the start box, drawn with Kaplan–Meier estimator. (b) Predicted exploration tendency (estimated marginal means) from GLMM with 75% confidence intervals for predicted values. Angling selection had opposing effects on exploration tendency in the two populations (Table 4). Predictions were made for the first trial repeat. For N in each group, see Table 1. Pink = HV, blue = LV

Fish emerged from the start box during the recorded time in ~84% of the trials. Mean latency for the fish that emerged was 1.83 min (range 0–9.54 min). There was a significant effect of angling selection (*p* = .027) and a slightly nonsignificant interaction effect (*p* = .054) of population background and angling selection on latency (Table 4). This was observed as an elevated risk to emerge in fish from LV background compared to HV background in the hatchery population, but not in the wild population (Figure 4a).

**TABLE 4:** Results of models for behavior traits in brown trout from hatchery and wild populations and two angling selection lines (HV and LV)

Exploration intensity (LMM)					
Fixed effects	Estimate ± *SE*	Num *df*	Res/Den *df*	*F*	*p*
Intercept	**0.30 ± 0.04**	**1**	**33.18**	**7.78 (*t*)**	**<.001**
Photoperiod	**−0.75 ± 0.04**	**3**	**84.03**	**6.53**	**.001**
Population	0.08 ± 0.06	3	42.50	1.40	.242
Selection	−0.01 ± 0.04	2	74.67	0.10	.903
Pop × selection	0.025 ± 0.06	1	197.41	1.28	.259
Photoperiod × pop	−0.070 ± 0.06	1	81.43	1.37	.245
Trial repeat	−0.017 ± 0.02	1	44.24	0.20	0.655

Random effects	Variance (*SD* ^2^)
ID	0.054^2^
Batch	0.036^2^
Arena	0.016^2^

Latency (frailty model)					
Fixed effects	Coef	*e* ^coef^	*SE*	*z*	*p*
Photoperiod	0.13	1.14	0.26	0.49	.630
Population	−0.083	0.92	0.30	−0.27	.780
Selection	**0.57**	**1.76**	**0.26**	**2.21**	**.027**
Pop × selection	−0.71	0.49	0.37	−1.92	.054
Photoperiod × pop	−0.060	0.94	0.38	−0.16	.870
Trial repeat	**0.23**	**1.26**	**0.082**	**2.83**	**.005**

Random effects	Variance (*SD* ^2^)
ID	0.625^2^
Batch	0.079^2^
Arena	0.097^2^

Exploration tendency (GLMM)				
Fixed effects	Estimate ± *SE*	Wald χ^2^	*df*	*p*
Intercept	0.55 ± 0.63	0.88 (*z*)	1	.380
Photoperiod	−0.63 ± 0.63	1.80	1	.179
Population	1.07 ± 0.72	0.058	1	.810
Selection	1.54 ± 0.64	0.43	1	.511
Pop × selection	**−2.46 ± 0.92**	**7.18**	**1**	**.007**
Photoperiod × pop	0.07 ± 0.88	0.006	1	.936
Trial repeat	**0.58 ± 0.21**	**7.79**	**1**	**.005**

Random effects	Variance (*SD* ^2^)
ID	1.398^2^
Batch	0.270^2^
Arena	0.762^2^

Note

The zero levels for contrasts in all models were as follows: photoperiod 12:12, population hatchery, and selection line HV. For model equations, see Table 2. For exploration intensity, *F*‐ and *p*‐values for the interactions and trial repeat were obtained from Type III test, and for the other main effects from linear hypothesis tests using restricted models with each main effect and its interactions set to zero. For latency, proportional hazard estimates for risk or emergence (± standard error) are shown with hazard ratios (*e* ^coef^). For latency and exploration tendency, Wald Chi‐square test was used to determine significance of fixed effects. Fixed effects with *p* < .05 shown in bold. For intercepts, *t* or *z* test values shown.

Exploration intensity did not differ between populations or angling selection lines, but the fish spent less time exploring the arena after acclimation in constant light compared to the 12 hr light:12 hr dark photoperiod (Table 4). Mean exploration intensity was 0.37 (range 0.0015–1.0).

Angling selection had contrasting effects on exploration tendency in each population: In the hatchery population, a higher proportion of fish from LV selection line were explorative than from HV selection line, while there was an opposite tendency in the wild population (Figure 4b; Table 4). In addition, exploration tendency increased with repeats of the behavioral trial.

Sex did not have a significant effect on any behavioral trait (female versus. male, Exploration intensity: *F*_1,39.612_ = 1.217, *p* = .277; Latency: *e* ^coef^ = 1.03, *z* = 0.29, *p* = .770; Exploration tendency: *z* = –0.514, *p* = .607).

There was no correlation between mass‐adjusted SMR and behavioral traits as assessed with initial, anticonservative test using individual BLUPs (see Houslay & Wilson, 2017) for neither exploration intensity (*r* = −0.006, *t* = −0.05_79_, *p* = .96) nor latency (*r* = −0.083, *t* = −0.75_79_, *p* = .46).

### 3.3 Behavioral responses to predator presence

There were no significant direct effects of predator cues on behavioral traits (Table 5). Repeating the behavior trials six times for each fish, three times with a burbot present and three times in control conditions, led to a significant decrease in exploration intensity with trial repeats (Table 5). The variance of exploration intensity between individuals appeared seemingly higher in the presence of burbot, but this was not significant in Levene's test of homogeneity of variance (*F*_1,93_ = 0.214, *p* = .645). Risk of emergence increased with increasing behavior trial repeats and between‐individual variation in latency was high (~10% higher variance in burbot versus control data compared to data from angling selection lines).

**TABLE 5:** Results of models for behavioral traits in the presence of predatory olfactory cues and control conditions in brown trout

Exploration intensity (LMM)				
Fixed effects	Estimate ± *SE*	Den *df*	*t*	*p*
Intercept	**0.435 ± 0.060**	**23.25**	**7.198**	**<.001**
Selection line	0.012 ± 0.065	9.59	0.18	.861
Treatment	−0.081 ± 0.042	15.85	−1.928	.072
Trial repeat	**−0.029 ± 0.010**	**71.62**	**−2.97**	**.004**
Body length	−0.00009 ± 0.004	8.63	−0.02	.984

Random effects	Variance (*SD* ^2^)
ID (burbot)	0.122^2^
ID (control)	0.077^2^
Batch	0.000
Arena	0.000
Residual	1.400^2^

Latency (frailty model)				
Fixed effects	Coef ± *SE*	*e* ^coef^	*z*	*p*
Selection line	0.336 ± 0.372	1.399	0.900	.370
Treatment	0.089 ± 0.220	1.093	0.400	.690
Trial repeat	0.113 ± 0.065	1.120	1.759	.080
Body length	−0.006 ± 0.026	0.994	−0.230	.820

Random effects	Variance (SD^2^)
ID	0.661^2^
Batch	0.210^2^
Arena	0.080^2^

Exploration tendency (GLMM)			
Fixed effects	Estimate ± *SE*	z	*p*
Intercept	1.449 ± 0.801	1.808	.0706
Selection line	0.659 ± 0.724	0.910	.363
Treatment	−0.63 ± 0.487	−1.294	.196
Trial repeat	−0.066 ± 0.142	−0.465	.642

Random effects	Variance (SD^2^)
ID	1.140^2^
Batch	0.000
Arena	(1.847 × 10^–5^)^2^

Note

For exploration intensity, the *t* test was used with Satterthwaite approximations to degrees of freedom and the model was fit with restricted maximum likelihood. For latency, proportional hazard estimates for risk or emergence (±*SE*) are shown with hazard ratios (*e* ^coef^). For latency and exploration tendency, Wald Chi‐square test was used to determine significance of fixed effects. The zero levels for contrasts in all models were as follows: treatment control and selection line HV. Significant effects (*p* < .05) shown in bold.

## 4 DISCUSSION

We found that captured and noncaptured brown trout produced offspring that differed in exploration‐related behaviors. Against our expectations, the LV selection line was more explorative in a new environment than the HV line in the hatchery population. However, in the wild population, there was no significant response to angling selection in exploration measured as latency to emerge. However, we found a significantly contrasting response in the likelihood of exploring the arena after emergence compared to the hatchery line, with the HV line being more explorative than the LV line. The results broadly agree with behavioral responses found in the offspring from the same angling selection experiment in their first summer, although the performed personality assays differed between the two studies; Alioravainen, Hyvärinen et al. (2020) assayed fingerlings at warm temperatures in simple arenas without predator cues. Together, these studies provide evidence for a heritable link between angling vulnerability and exploratory behavior assessed in controlled settings but highlight that such connections can be population or environment specific. The smaller differences in the behavior between angling selection lines from the wild population than from the hatchery population may result from several reasons. First, genetic variation in the wild population was smaller than in the hatchery population (Lemopoulos, Prokkola et al., 2019). Second, the parental wild fish had no juvenile hatchery history, and they were captured directly from seminatural ponds with a stream and a pool section during angling, while the hatchery fish were fished in a plain concrete tank under high density, which may have introduced density‐dependent behavioral effects that were absent in the wild fish.

The result that mass‐corrected SMR was higher in the offspring of wild fish than those from hatchery parents was unexpected. The hatchery environment is expected to favor individuals with a fast metabolic rate (Robertsen et al., 2019). However, because metabolic rate measurements can induce stress responses (Murray et al., 2017) and domestication‐related phenotypic changes can be evident in hatchery environments already after a single generation in salmonids (Christie et al., 2016; Islam et al., 2020), the result could be explained by a better ability of the hatchery‐population fish to adjust to handling during metabolic rate measurements. Further, metabolic rate might relate to the life‐history strategy of the fish (Rosenfeld et al., 2015), as the wild fish represented resident and the captive fish migratory brown trout forms (Lemopoulos et al., 2019). The potential effects of angling selection on metabolic rate were smaller than the effects of population, and our technique lacked sensitivity to detect minor differences between groups, but this could be reassessed in further studies.

Differences in stress coping styles, that is, sensitivity of the neuroendocrine stress responses (Koolhaas et al., 2010; Schjolden et al., 2005), could partly explain why the results on behaviors and SMR were partly contradicting our expectations. If the LV fish from the hatchery population were more reactive compared to the HV fish, their behavior may have indicated a higher stress response to the experiment and heightened escape behavior; this effect has also been suggested to occur in pike (*Esox lucius*) (Laskowski et al., 2016). Angling intensity itself can also influence the serotonergic and dopamine systems in fish (Koeck et al., 2018), and given that Koeck et al. (2018) found clear species differences in the effect between rainbow trout and brown trout, it can be speculated that population differences in how fish respond physiologically to disturbance caused by angling are also possible. Changes in stress‐related physiological responses could explain which individuals are most vulnerable to angling. Relatedly, the most angling‐vulnerable parent fish in the hatchery strain may have had the lowest status in the dominance hierarchy within the concrete ponds, and therefore been the hungriest and the most prone to attack the fly patterns. In contrast to the hatchery population, wild fish were under more natural‐like ponds during the angling trials and this may have allowed to them to maintain more of their natural behavior, including hierarchy related to feeding, and by extension, to attacking the flies.

It is likely that for both population and angling selection line differences that genetic inheritance would explain our results at least partly; Ågren et al. (2019) showed that the heritability in exploration‐related principal component was 0.1 ± 0.065 *SE* in the same hacthery strain of brown trout, along with hatchery‐wild crossbred strains. In Kortet et al. (2014), low heritabilities were reported for exploration and boldness‐related personality axis, but that of freezing tendency was 0.14, similar to Ågren et al. (2019). In three‐spined stickleback (*Gasterosteus aculeatus*), behavioral traits related to exploration and boldness in the presence and absence of predation risk were moderately heritable (Dingemanse et al., 2009). Importantly, angling vulnerability itself is heritable in largemouth bass (Philipp et al., 2009).

Photoperiod had no influence on the relative differences between populations or on the absolute metabolic and behavioral traits, except on exploration intensity, which, however, was not affected by population background or angling selection. This suggests that exploration and metabolic rate differences between populations can be consistent across environments. Constant light is not encountered by brown trout during the winter; hence, the 24‐hr light regime could be considered unnatural and potentially stressful for the fish. Constant light can disrupt entrainment of endogenous rhythms by inhibiting the synthesis of melatonin and by directly affecting photosensitive proteins (Falcón et al., 2010; Peirson et al., 2009). In general, nontropical species are expected to be particularly sensitive to photoperiod disturbances due to the role of day length in anticipating seasonal changes in environmental conditions (Borniger et al., 2017).

The differences we found between populations can also be explained by the level of domestication, as the hatchery stock had been reared in captivity for several generations. Populations frequently differ in, for example, metabolic rate and behavioral syndromes (Dingemanse et al., 2007; Lahti et al., 2002; Polverino et al., 2018), driven by environmental differences, natural selection, founder effects, and genetic drift. The populations we studied also differed in their life histories, with the wild population being clearly less migratory than the hatchery population (Lemopoulos et al., 2019). Moreover, juveniles from the wild population show lower tendency for postrelease dispersal in a stream environment than juveniles from the hatchery population (Alioravainen, Prokkola et al., 2020). Although we reared offspring under common garden conditions and maximized genetic diversity within each group through a fully factorial breeding matrix, it is possible that differences in the early rearing environments of wild and hatchery parents had contrasting effects on offspring through parental or epigenetic effects (Crews et al., 2012; Reddon, 2012). The duration of these effects on offspring physiology can be short or long lasting (Bell et al., 2016; Metzger & Schulte, 2016; Munday et al., 2017).

Our goal was to study exploration/risk‐taking‐related behaviors by subjecting fish to the olfactory cues of a natural predator that had fed on conspecifics, which was expected to cause a strong antipredator response (Ferrari et al., 2010; Vilhunen & Hirvonen, 2003). Although no direct effect of predator presence on brown trout behavior was found in this study, we found a strong decline in the exploration intensity of fish with increasing predator/control trial repeats, which could indicate the development of an antipredator response, evident as increased hiding and decreased activity in juvenile salmonids (Kopack et al., 2015; Vilhunen, 2006; Vilhunen & Hirvonen, 2003). However, we did not separate fish into control and predator exposure treatments, but each fish in the test served as their own control, and thus, the effect may also be related to habituation into the test arenas. We conducted the test of predator cues only on the wild fish offspring, given that innate responses were expected to be stronger in this population, but notably none of the individuals in the behavior trials in this study had been exposed to predators before the trials, apart from potential traces of piscivore odors in the rearing water. It is therefore not surprising that results were not as strong as in previous studies using wild‐caught individuals (Álvarez & Nicieza, 2003). In our study, the scarcity of responses to the presence of predator odor, measured in the offspring of wild fish, indicate only weak innate responses. Nevertheless, the (nonsignificant) tendency for lower exploration intensity in the presence of burbot than in control conditions resembles previously shown antipredator responses in salmonids.

## 5 CONCLUSIONS

Our results suggest population‐specific potential for rapid human‐induced evolution in the behavior of a popular fishing target species. Population differences in the response to selection may have arisen from contrasting dependence of angling vulnerability from fish behavior, or from differences in the heritability of selected behaviors. This highlights the complexity of ecological and innate factors that can contribute to angling‐induced selection in natural populations. Overall, the results from this common garden experiment suggest a significant genetic effect upon the behavior of brown trout parr.

## CONFLICT OF INTEREST

The authors declare that they have no conflict of interest.

## AUTHOR CONTRIBUTION

**Jenni M. Prokkola:** Conceptualization (equal); Data curation (equal); Formal analysis (equal); Investigation (equal); Methodology (equal); Project administration (equal); Visualization (lead); Writing‐original draft (lead); Writing‐review & editing (lead). **Nico Alioravainen:** Conceptualization (supporting); Formal analysis (supporting); Investigation (equal); Methodology (equal); Writing‐original draft (equal); Writing‐review & editing (supporting). **Lauri Mehtätalo:** Formal analysis (equal); Writing‐original draft (supporting); Writing‐review & editing (supporting). **Pekka Hyvärinen:** Conceptualization (supporting); Funding acquisition (supporting); Investigation (supporting); Resources (equal); Supervision (supporting); Writing‐original draft (supporting); Writing‐review & editing (supporting). **Alexandre Lemopoulos:** Investigation (supporting); Methodology (supporting); Writing‐original draft (supporting); Writing‐review & editing (supporting). **Sara Metso:** Investigation (supporting); Methodology (supporting); Writing‐original draft (supporting); Writing‐review & editing (supporting). **Anssi Vainikka:** Conceptualization (lead); Formal analysis (supporting); Funding acquisition (lead); Investigation (supporting); Methodology (supporting); Project administration (supporting); Resources (supporting); Software (equal); Supervision (lead); Writing‐original draft (supporting); Writing‐review & editing (supporting).

## Supporting information

Appendix S1

## Data Availability

Raw respirometry data and behavioral data are available in Dryad (https://doi.org/10.5061/dryad.crjdfn336). All raw data and R codes for analyzing respirometry data and for the statistical analysis are also available in GitHub (https://github.com/jprokkola/Strutta_repo). Videos of behavior trials are available in Figshare (https://doi.org/10.6084/m9.figshare.7938173.v1 and https://doi.org/10.6084/m9.figshare.7932332.v1).
